# Cemento-ossifying fibroma: A South American multicenter study

**DOI:** 10.4317/medoral.80037

**Published:** 2026-03-01

**Authors:** Flavio Veiga do Pilar Cobra, Éricles Otávio Santos, Mariana Villarroel Dorrego, Mônica Simões Israel, Claudia Patricia Peña-Vega, Juan Pablo Rodríguez-Mora, Alejandra Rojas Ponte, Ricardo Luiz Cavalcanti de Albuquerque-Junior, Rogério Gondak, Amanda Claudino Gomes, Pollianna Muniz Alves, Daniel Santiago Vale, Diego Antônio Costa Arantes, Elismauro Francisco Mendonça, Layanne Ferreira Ribeiro e Sobral, Bárbara Mendes de Jesus, Hercílio Martelli-Júnior, Lucas Guimarães Abreu, Victor Zanetti Drumond, Sameep S Shetty, Maria Luísa Gomes Souza, Gerhilde Callou Sampaio, José Alcides Almeida de Arruda, Jefferson R Tenório, Ricardo Alves Mesquita, Bruno Augusto Benevenuto de Andrade

**Affiliations:** 1 Department of Oral Diagnosis and Pathology, School of Dentistry, Universidade Federal do Rio de Janeiro, Rio de Janeiro, Brazil; 2 School of Dentistry, Universidad Central de Venezuela, Caracas, Venezuela; 3 Department of Diagnosis and Therapeutics, School of Dentistry, Universidade do Estado do Rio de Janeiro, Rio de Janeiro, Brazil; 4 Department of Oral Medicine, Faculdade São Leopolod Mandic, Brazil; 5 Department of Basic Science and Oral Medicine, School of Dentistry, Universidad Nacional de Colombia, Bogotá, Colombia; 6 Sanitas-Keralty, Servicio de Cirugía Oral y Maxilofacial, Bogotá, Colombia; 7 Department of Pathology, Health Sciences Center, Universidade Federal de Santa Catarina, Florianópolis, Brazil; 8 Department of Dentistry, Universidade Estadual da Paraíba, Campina Grande, Brazil; 9 Department of Stomatology (Oral Pathology), School of Dentistry, Universidade Federal de Goiás, Goiânia, Brazil; 10 Department of Oral Pathology and Oral Medicine, School of Dentistry, Universidade Estadual de Montes Claros, Montes Claros, Brazil; 11 Department of Child and Adolescent Oral Health, School of Dentistry, Universidade Federal de Minas Gerais, Belo Horizonte, Brazil; 12 Department of Oral Surgery, Pathology and Clinical Dentistry, School of Dentistry, Universidade Federal de Minas Gerais, Belo Horizonte, Brazil; 13 Manipal College of Dental Sciences, Mangalore, Manipal Academy of Higher Education, Manipal, India; 14 School of Medicine, Afya Centro Universitário, Montes Claros, Brazil; 15 Department of Oral and Maxillofacial Surgery and Pathology, School of Dentistry, Universidade de Pernambuco, Recife, Brazil

**Keywords:** Bone, cemento-ossifying fibroma, jaw, odontogenesis, odontogenic tumor, oral and maxillofacial pathology.

## Abstract

**Background:**

Cemento-ossifying fibroma (COF) is a benign odontogenic fibro-osseous neoplasm with the proliferation of a cellular fibrous stroma. This study aimed to characterize the clinicodemographic, radiographic, and therapeutic profile of COF in Brazil, Colombia and Venezuela.

**Materials and Methods:**

Eight oral pathology and oral medicine services participated. Clinical, radiographic and treatment-related data were retrieved from patient records. Diagnoses were established according to the 5th edition of the WHO classification of odontogenic and maxillofacial bone tumors. Data were analyzed descriptively.

**Results:**

A total of 112 cases were included; 44.6% were from Brazil and 27.7% each from Venezuela and Colombia. Most individuals were female (n=97; 86.6%), with a mean age of 35.6±12.6 years. Posterior mandibular involvement predominated (n=47; 65.3%). Mixed radiographic patterns were most frequent (n=46; 50.0%), and cortical expansion was observed in 52.7% (n=48). Complete surgical removal of the lesion without prior incisional diagnosis and enucleation/curettage were the most common management approaches (n=69; 61.6%). Recurrence occurred in 8.3% of individuals with the available follow-up.

**Conclusions:**

The study offers the first multicenter documentation of COF in South America, corroborating prior literature regarding its predilection for women in the third to fourth decades of life and for posterior mandibular sites. Subtle clinicodemographic variants were noted across the three countries.

## Introduction

Cemento-ossifying fibroma (COF) is a benign odontogenic fibro-osseous neoplasm of the jaws characterized by the proliferation of a cellular fibrous stroma containing variable amounts of woven bone, lamellar bone and cementum-like calcifications [1,2]. In South America, COF accounts for approximately 5.8% of all odontogenic tumors [3]. Although a mandibular fibro-osseous lesion compatible with what is now recognized as COF was first described by Menzel in 1842, and the term ossifying fibroma was later introduced by Montgomery in 1927 [4], the terminology has been revised over the subsequent years. Historically, COF has been described using other designations, including cementifying fibroma, ossifying fibroma, ossifying-odontogenic fibroma, and periodontoma [5].

Current World Health Organization (WHO) classification of odontogenic and maxillofacial bone tumors places COF within the group of benign mesenchymal odontogenic tumors, distinguishing it from the heterogeneous category of fibro-osseous tumors and dysplasias [6]. Central to this distinction is the long-standing question of etiopathogenesis. Traditionally, COF was presumed to arise from multipotent mesenchymal cells of the periodontal ligament, given its restriction to tooth-bearing regions and its capacity to generate cementum-like material [7]. Nevertheless, direct evidence supporting a periodontal ligament origin remained elusive, particularly after reports of microscopically identical lesions occurring in the frontal, temporal, sphenoid, and ethmoid bones challenged this assumption [8]. Additionally, a proteomic comparison of COF and fibrous dysplasia revealed differential expression of matricellular proteins involved in osteoblastic differentiation and mineralization, underpinning the concept of distinct underlying pathogenetic mechanisms [9].

Clinically and radiographically, COF demonstrates substantial heterogeneity, frequently overlapping with benign fibro-osseous lesions [10]. It typically presents as an asymptomatic swelling, most often occurring in the mandible [11,12]. Radiographically, COF most commonly appears as a well-circumscribed unilocular radiolucency that gradually develops central opacification as mineralized material accumulates [5,13,14]. Larger lesions may lead to cortical expansion, displacement of adjacent anatomical structures, and, less frequently, root resorption [13]. However, these features are not pathognomonic and may also emulate odontogenic cysts and tumors, making accurate diagnosis reliant on clinical, surgical, radiographic, and microscopic correlation [7,15].

Despite the extensive literature on fibro-osseous lesions [1,7,10], epidemiological data specific to COF remain scarce, particularly from South America. A recent systematic review demonstrated that approximately 60% of published studies originate from Asian countries, especially India [2]. Apparently, three earlier multicenter studies have been published hitherto; one that included exclusively COF cases [16], and two others that included samples of juvenile ossifying fibroma (JOF) [17,18]. The aim of the present multicenter series was to describe COF cases diagnosed in Brazil, Colombia, and Venezuela, providing an overview of data on clinicodemographic and radiographic characteristics as well as information on treatment and recurrence.

## Materials and Methods

*Sampling, setting and ethical clearance*

The series comprised a convenience sample of 112 histopathologically confirmed COF cases, collected between 2021 and 2025 from the archives of eight oral pathology and oral medicine services across South America. Cases were contributed by Universidad Nacional de Colombia, Bogotá, Colombia (n=31); Universidad Central de Venezuela, Caracas, Venezuela (n=31); Universidade Federal de Goiás, Goiânia (n=21); Universidade Federal de Santa Catarina, Florianópolis (n=17); Universidade Federal de Minas Gerais, Belo Horizonte (n=4); Universidade do Estado do Rio de Janeiro, Rio de Janeiro (n=4); Universidade Estadual de Montes Claros, Montes Claros (n=2); and Universidade Estadual da Paraíba, Campina Grande (n=2), the latter institutions all located in Brazil. The study was approved by the Research Ethics Committees (18-23/57829, 42095715.1.0000.0121 and 10723019.0.1001.5149) and Material Transfer Agreements were drafted and signed to formalize the collaborative framework. In accordance with the principles of the Declaration of Helsinki, participant anonymity and data confidentiality were strictly maintained.

*Data collection*

Data were extracted from information provided by the clinician on patients’ records, as follows: Gender, age, evolution time (months), anatomical location (anterior: Lesions in the incisor/canine region; posterior: Lesions in the premolar, molar, retromolar, ramus, or maxillary sinus region), symptomatology (e.g., pain, swelling), radiographic characteristics (radiolucent, mixed, or radiopaque; and unilocular or multilocular), cortical bone alteration (expansion and/or perforation), association with teeth (impaction, tooth displacement, and/or tooth root resorption), lesion size (mm), treatment, and recurrence.

*Diagnostic rendering*

From each paraffin block, a 4-μm section was stained with hematoxylin and eosin. Diagnoses were rendered by experienced oral and maxillofacial pathologists. Diagnoses were established according to the 5th edition of the WHO classification of odontogenic and maxillofacial bone tumors [6]. Histopathologically, COF was identified by a well-demarcated benign fibro-osseous tissue composed of a cellular fibrous stroma and variable mineralized components, including woven to cancellous bone, osteoid, and cementum-like calcifications, with occasional osteoblastic rimming and low stromal vascularity. Cases exhibiting psammomatoid or trabecular patterns, syndromic conditions, gnathodiaphysial dysplasia, synchronous lesions, or hybrid odontogenic lesions were excluded.

*Data analysis*

Data were tabulated in Microsoft Office Excel 2019 (Microsoft, Redmond, WA, USA) and subjected to descriptive analysis in GraphPad Prism version 8.0.0 for Windows (GraphPad Software, San Diego, CA, USA), using mean±standard deviation, counts, and percentages.

## Results

*Clinicodemographic features*

A total of 112 cases were included in the study, among whom 50 (44.6%) were from Brazil, 31 (27.7%) from Venezuela, and 31 (27.7%) from Colombia. Detailed information about the cases of each country is reported in Supplementary Table 1 (https://www.medicinaoral.com/medoralfree01/28061/Suppl1.pdf). Ninety-seven (86.6%) individuals were female and 15 (13.4%) were male, yielding a female-to-male ratio of 6.5:1. The most represented age group was 40-49 years (n=31; 28.2%) (Figure 1A). The mean age of the sample was 35.6±12.6 years (range: 10-64 years); the mean age of women was 36.6 years and of men was 29.7 years. The mean duration of lesions was 21.8±20.6 months (range: 1-120 months).

Regarding symptomatology, swelling was reported in 48 (44.4%) individuals, followed by pain in 18 (16.7%). Twenty-eight (25.9%) individuals were asymptomatic (Figure 1B). The mandible was the most frequently affected site (n=72; 64.3%), particularly the posterior region (n=47; 65.3%) (Figure 2). The mean lesion size was 28.2±21.9mm (range: 1-120mm) (Figure 1C). Representative clinical and radiographic aspects of COF lesions are illustrated in Figure 3.

*Radiographic features*

Radiographically, most lesions exhibited a mixed pattern (n=46; 50.0%), with cortical expansion as the most common alteration (n=48; 52.7%) and a predominantly unilocular internal appearance (n=37; 78.7%). Most cases had no tooth involvement (n=35; 31.8%). Among cases with tooth alterations, the most frequent change was tooth displacement (n=33; 30.0%) (Figure 1D-G). Data on specific tooth involvement were available for 101 cases. The tooth most frequently affected was the mandibular right second premolar (n=11; 10.9%), followed by the mandibular right first molar and mandibular right canine (n=9; 8.9% each).

*Management*

Regarding management, complete surgical removal of the lesion without prior incisional diagnosis was performed in 35 (31.2%) cases, followed by enucleation/curettage in 34 (30.3%) cases (Figure 1H). Incisional biopsy was performed in 30 (26.8%) cases; however, the final treatment modality was undetermined. Histopathologically, the lesions exhibited a classic fibro-osseous architecture composed of a moderately cellular fibrous stroma interspersed with trabeculae of woven to lamellar bone and cementum-like calcifications. Occasional osteoid deposition and areas of stromal hypercellularity without pleomorphism were also observed (Figure 4). Recurrence data were available for 60 individuals; of these, 55 (91.7%) showed no recurrence and five (8.3%) experienced recurrences, with intervals ranging from one to nine years (Figure 1I).

## Discussion

We provide novel multicenter data on 112 COF cases from three South American countries, corroborating key epidemiologic patterns described in classical studies while also revealing noteworthy regional distinctions. Consistent with the seminal clinicopathologic analysis by Eversole *et al*. [13], this series demonstrated a marked female predominance, a concentration in the third to fourth decades of life, and a clear preference for the molar-premolar region of the mandible; findings that align with previous literature reporting female-to-male ratios of approximately 5:1 [1,6,10,19]. However, when disaggregated by country, the data revealed more nuanced demographic trends. Venezuelan cases occurred in comparatively younger individuals, with a greater proportion of adolescents and young adults, and exhibited a less pronounced female predominance (2.9:1) when compared to Brazil (9:1) and Colombia (14.5:1). These differences suggest that gender and age distributions may vary across South American populations, potentially reflecting divergent referral pathways, disparities in access to specialized care, and broader structural heterogeneity within regional health systems [20].

Geographic variation in fibro-osseous lesions has been documented elsewhere [1,7,12], although meaningful comparisons across series remain challenging due to evolving WHO criteria and shifts in diagnostic nomenclature over time [7]. Historically, COF was subsumed within the broad fibro-osseous category, leading many earlier investigations to report its frequency relative to all fibro-osseous lesions, i.e., typically ranging from 24.3% to 32.4% [12,15]. Nevertheless, with refinements introduced in the 4th and, more decisively, the 5th edition of the WHO classification, COF is now formally delineated as a benign mesenchymal odontogenic tumor. Consequently, contemporary epidemiological surveys adhering to WHO 5th-edition diagnostic standards report substantially lower relative frequencies, with COF comprising approximately 4.0-5.8% of all odontogenic tumors [3,21]. On the one hand, these taxonomic and conceptual shifts underscore the need for caution when comparing studies across decades, populations, and diagnostic eras; on the other hand, they highlight how earlier fibro-osseous datasets may have inadvertently aggregated biologically and histogenetically distinct entities now recognized as unrelated to COF [7].

The predominance of premolar-molar mandibular lesions in this series is fully aligned with classic descriptions of COF topography [5,13,14,19]. In this region, COF overlaps anatomically and radiographically with a broad spectrum of fibro-osseous and odontogenic entities (e.g., cemento-osseous dysplasia, fibrous dysplasia, JOF, ameloblastoma, odontogenic keratocyst, and central giant cell granuloma), each capable of presenting as well-circumscribed radiolucent or mixed lesions that complicate diagnostic accuracy [11,22,23]. In this line, we reinforce the key observation of Vieira *et al*. [24], who stated that posterior mandibular lesions are disproportionately vulnerable to diagnostic misclassification, with odontogenic cysts and tumors and even malignancies occasionally mistaken for endodontic or fibro-osseous processes. A systematic review has likewise highlighted the persistent inconsistency in radiographic differentiation among fibro-osseous lesions [2], emphasizing the need for meticulous clinicoradiographic-histopathologic correlation. Although most COF in the present series adhered to the canonical posterior mandibular distribution, approximately 18% involved the anterior maxilla, underscoring that, while topographically characteristic, COF is not site-restricted and should remain in the differential diagnosis when mixed or radiolucent lesions arise in anatomically atypical regions.

Radiographically, our series corroborates the classical evolution of COF from well-defined radiolucent lesions to mixed and, occasionally, predominantly radiopaque masses as mineralized tissue accumulates. In the present study, mixed lesions were the most frequent pattern, aligning with previous series and reviews that describe a continuum from purely radiolucent to densely sclerotic appearances, often within the same lesion over time [10,13,14]. The high frequency of cortical expansion observed in our cases, particularly within the Brazilian subset, is likewise consistent with the characterization of COF as a well-demarcated expansile lesion capable of producing cortical thinning, tooth displacement, and, less commonly, root resorption. Tooth displacement emerged as the predominant dentoalveolar change, whereas root resorption was relatively infrequent, in agreement with former studies [14,19]. Of note, our research group has evaluated CBCT-based quantitative methods and fractal analysis, demonstrating that COF and other fibro-osseous lesions exhibit measurable textural and structural differences on advanced imaging, thereby underscoring the potential of refined radiologic metrics to enhance diagnostic confidence [12,25]. In this regard, COF is indistinguishable using histology or imaging alone, reaffirming the necessity of rigorous clinicoradiographic-pathologic correlation for a correct diagnosis [3,10,24].

Accumulating molecular and clinicopathologic evidence continues to refine the conceptualization of COF as a bona fide odontogenic neoplasm. Ide *et al*. [26] provided compelling radiologic, surgical, and microscopic documentation of a COF arising directly from the lateral periodontal ligament, including preserved rests of Malassez; an observation that strongly supports a periodontal-ligament origin for at least a subset of central COF. This contrasts with ossifying fibromas reported in craniofacial bones lacking any anatomical relationship to the periodontal ligament, suggesting that the broader fibro-osseous tumor family likely encompasses both odontogenic and non-odontogenic subtypes [8]. Molecular studies further reinforce this biological distinction. COF lacks the GNAS mutations and exhibits CDC73 alterations in only a minority of sporadic and syndromic cases [27,28]. Histopathologically, COF is characterized as a well-demarcated lesion composed of a moderately cellular fibroblastic stroma containing woven-to-lamellar bone, osteoid, and basophilic cementum-like calcifications, often accompanied by osteoblastic rimming and centripetal maturation. Intralesional variability is common, and the typically low mitotic activity and scant vascularity may overlap with features seen in psammomatoid or trabecular ossifying fibromas, underscoring the potential for microscopic similarity among these entities [6,29].

The treatment modalities recorded in this series are consistent with current practice recommendations for COF [18,30]. Enucleation/curettage was the most common approach, whereas more extensive resections were reserved for a minority of cases in which lesions were larger or associated with cortical perforation. This aligns with the literature indicating that COF typically “shells out” readily and can be managed conservatively, with rare recurrences when adequately removed, and is supported by earlier outcome studies reporting low recurrence after conservative surgery [18,30]. The recurrence rate of 8.3% among individuals with follow-up falls within the broad range described in the literature, where recurrence varies from near-zero to approximately 20-25% depending on case mix, duration of surveillance, and inclusion of juvenile variants [13,18]. Considering the limited follow-up information for about half of the study cohort, the true recurrence rate is unknown; nevertheless, our findings reinforce the concept that COF carries a generally favorable prognosis when treated appropriately. It is important to mention that in some centers, biopsy procedures preceded referral to tertiary units for definitive surgery and follow-up; information that was unavailable in some records.

Limitations of the present study should be acknowledged. First, the retrospective design and reliance on archival records inherently restricted data completeness, particularly for radiographic descriptors and follow-up information. Second, imaging protocols were not standardized across centers; many cases were evaluated with the conventional panoramic radiographs, with variable access to computed tomography, which may have refined the detection of specific radiographic details. Third, although diagnoses were rendered by experienced oral and maxillofacial pathologists in accordance with WHO, the incorporation of ancillary tests (e.g., molecular assays) was unfeasible. Despite these constraints, the study has strengths. It compiles a comparatively large number of COF cases from eight academic services across three countries (Brazil, Colombia, and Venezuela) and applies contemporary WHO diagnostic criteria that enable meaningful multicenter comparison. However, the findings should not be interpreted as fully representative of the entire South American population. Differences in healthcare systems, referral patterns, and demographic structures across other South American countries may limit the generalizability of these data.

## Conclusions

In summary, this multicenter South American series contributes to the epidemiologic and clinico-radiographic understanding of COF, reaffirming its female predominance, typical onset in early to mid-adulthood, and preference for the posterior mandible. It also reveals subtle regional variations across Brazil, Colombia and Venezuela that warrant further investigation. Future studies incorporating molecular profiling and long-term follow-up are needed to filter the diagnostic accuracy and optimize management across diverse populations.

## Figures and Tables

**Table 1:** Table 1. Data on clinicodemographic, treatment, and recurrence of individuals with cemento-ossifying fibroma in South America, disaggregated by country (n=112),

*Variable*	*Brazil (n=50, %)*	*Colombia (n=31, %)*		*Venezuela (n=31, %)*
**Sex**				
Male	5 (10.0)	2 (6.5)	8 (25.8)	
Female	45 (90.0)	29 (93.5)	23 (74.2)	
Ratio	9:1	14.5:1	2.9:1	
**Age, mean (±SD), range**				
General	37.2 (±13.1), 10-64	39.2 (±10.1), 22-59	29.5 (±11.9), 12-49	
Male	33.8 (±13.9), 18-52	29 (±6), 23-35	26.1 (±14.2), 12-49	
Female	37.6 (±12.9), 10-64	39.9 (±9.9), 22-59	30.7 (±10.7), 12-49	
**Age, decades of life**				
10-19	5 (10.0)	-	8 (25.8)	
20-29	9 (18.0)	5 (16.1)	8 (25.8)	
30-39	12 (24.0)	11 (35.5)	6 (19.4)	
40-49	14 (28.0)	8 (25.8)	9 (29.0)	
50-59	7 (14.0)	6 (19.4)	-	
60-69	2 (4.0)	-	-	
NI	1 (2.0)	1 (3.2)	-	
**Evolution time, mean (±SD), range (months)**	19.4 (±17.2), 2-84	32.9 (±30.8), 4-120	19.4 (±15.0), 1-48	
**Clinical features/symptomatology** ^*^				
Asymptomatic	9 (15.5)	10 (30.3)	9 (28.1)	
Swelling	28 (48.3)	9 (27.3)	11 (34.4)	
Painful	4 (6.9)	2 (6.1)	12 (37.5)	
Painless	7 (12.0)	-	-	
Slow growing	3 (5.2)	-	-	
Paresthesia	3 (5.2)	-	-	
Nasal congestion	-	1 (3.0)	-	
NI	4 (6.9)	11 (33.3)	-	
**Anatomical location**				
Anterior maxilla	18 (36.0)	-	2 (6.5)	
Posterior maxilla	6 (12.0)	4 (12.9)	5 (16.1)	
Maxilla (unspecified)	3 (6.0)	1 (3.2)	-	
Anterior-posterior maxilla	1 (2.0)	-	-	
Anterior mandible	8 (16.0)	6 (19.4)	6 (19.3)	
Posterior mandible	12 (24.0)	17 (54.8)	18 (58.1)	
Anterior-posterior mandible	2 (4.0)	3 (9.7)	-	
**Radiographic appearance**				
Radiolucent	17 (34.0)	8 (25.8)	11 (35.5)	
Radiopaque	7 (14.0)	1 (3.2)	2 (6.4)	
Mixed	18 (36.0)	10 (32.3)	18 (58.1)	
NI	8 (16.0)	12 (38.7)	-	
**Internal radiographic pattern**				
Unilocular	12 (24.0)	8 (25.8)	17 (54.9)	
Multilocular	4 (8.0)	1 (3.2)	5 (16.1)	
NI	34 (68.0)	22 (71.0)	9 (29.0)	
**Cortical bone alteration** ^*^				
Expansion	34 (64.1)	4 (12.9)	10 (32.2)	
Perforation	6 (11.3)	6 (19.4)	6 (19.4)	
No alteration	8 (15.1)	2 (6.4)	15 (48.4)	
NI	5 (9.5)	19 (61.3)	-	
**Tooth involvement** ^*^				
Yes (unspecified)	8 (15.7)	19 (55.9)	-	
No	6 (11.8)	10 (29.4)	19 (61.3)	
Tooth displacement	25 (49.0)	-	8 (25.8)	
Tooth mobility	1 (2.0)	5 (14.7)	-	
Tooth impacted	1 (2.0)	-	-	
Root resorption	4 (7.8)	-	4 (12.9)	
NI	6 (11.7)	-	-	
**Lesion size, mean (±SD), range (mm)**	25.4 (±22.1), 1-100	24.9 (±26.0), 3-120	34.4 (±17.2), 10-80	
**Treatment**				
Complete surgical removal of the lesion without prior incisional diagnosis	13 (26.0)	22 (71.0)	-	
Incisional biopsy	23 (46.0)	7 (22.6)	-	
Enucleation/curettage	13 (26.0)	-	21 (67.7)	
Block resection	-		7 (22.6)	
Segmental resection	1 (2.0)		3 (9.7)	
Hemimandibulectomy	-	2 (6.4)	-	
**Recurrence**				
Yes	4 (8.0)	-	1 (3.2)	
No	25 (50.0)	-	30 (96.8)	
NI	21 (42.0)	31 (100.0)	-	

Note: NI, not informed; SD, standard deviation. *These variables were analyzed not by the number of individuals, but by the number of manifestations; consequently, a single individual may have exhibited more than one manifestation.

**Figure 1:**
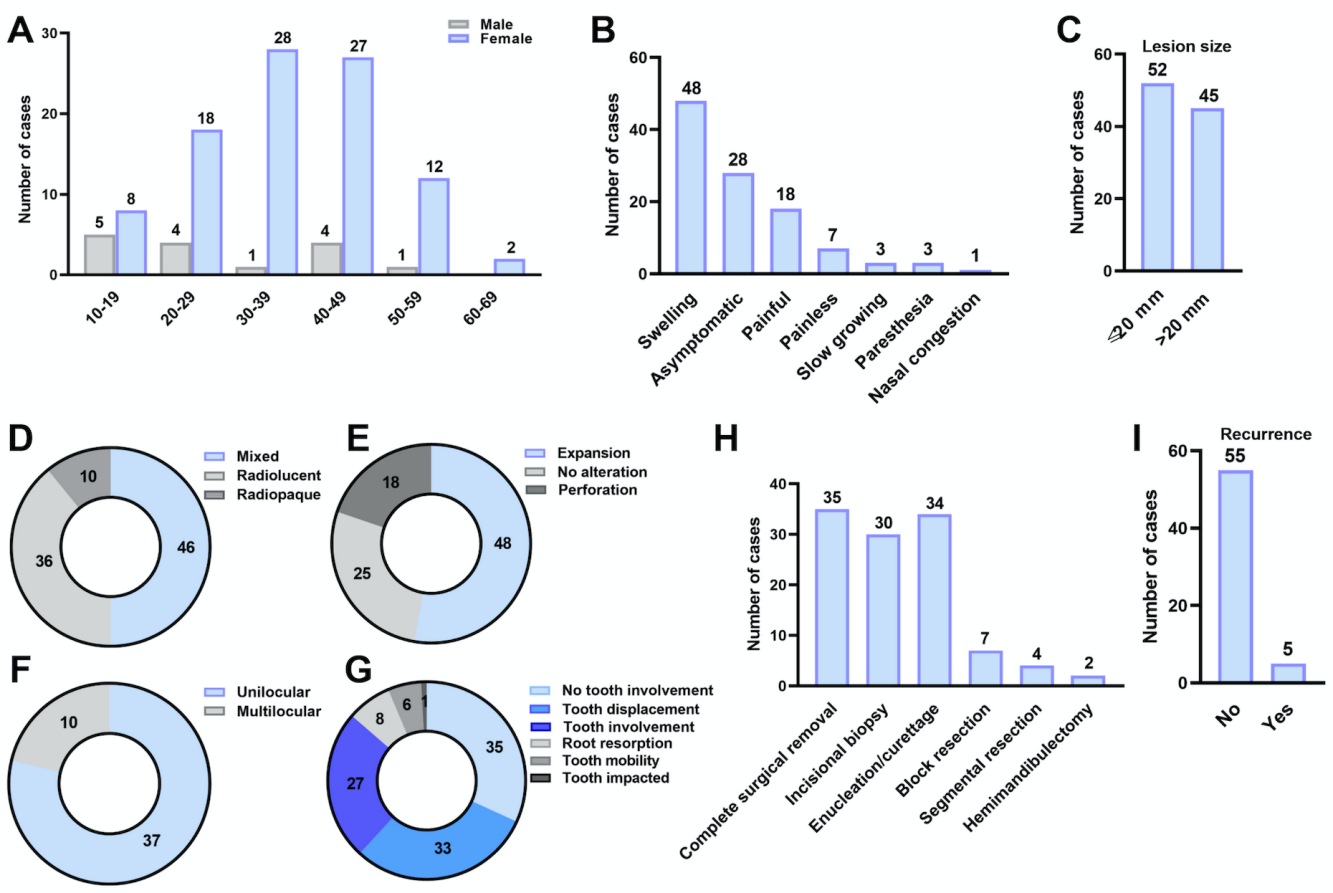
Fig. 1: Distribution of cemento-ossifying fibroma cases. (A) Age distribution by gender, (B) clinical presentations/symptomatology, (C) lesion size stratification, (D) radiographic appearance, (E) cortical bone alteration, (F) internal radiographic pattern, (G) tooth involvement, (H) management, and (I) recurrence. Note: The unit of analysis for the variables clinical presentations, cortical bone alteration, and tooth involvement was not the number of individuals, as more than one feature could occur in the same case.

**Figure 2:**
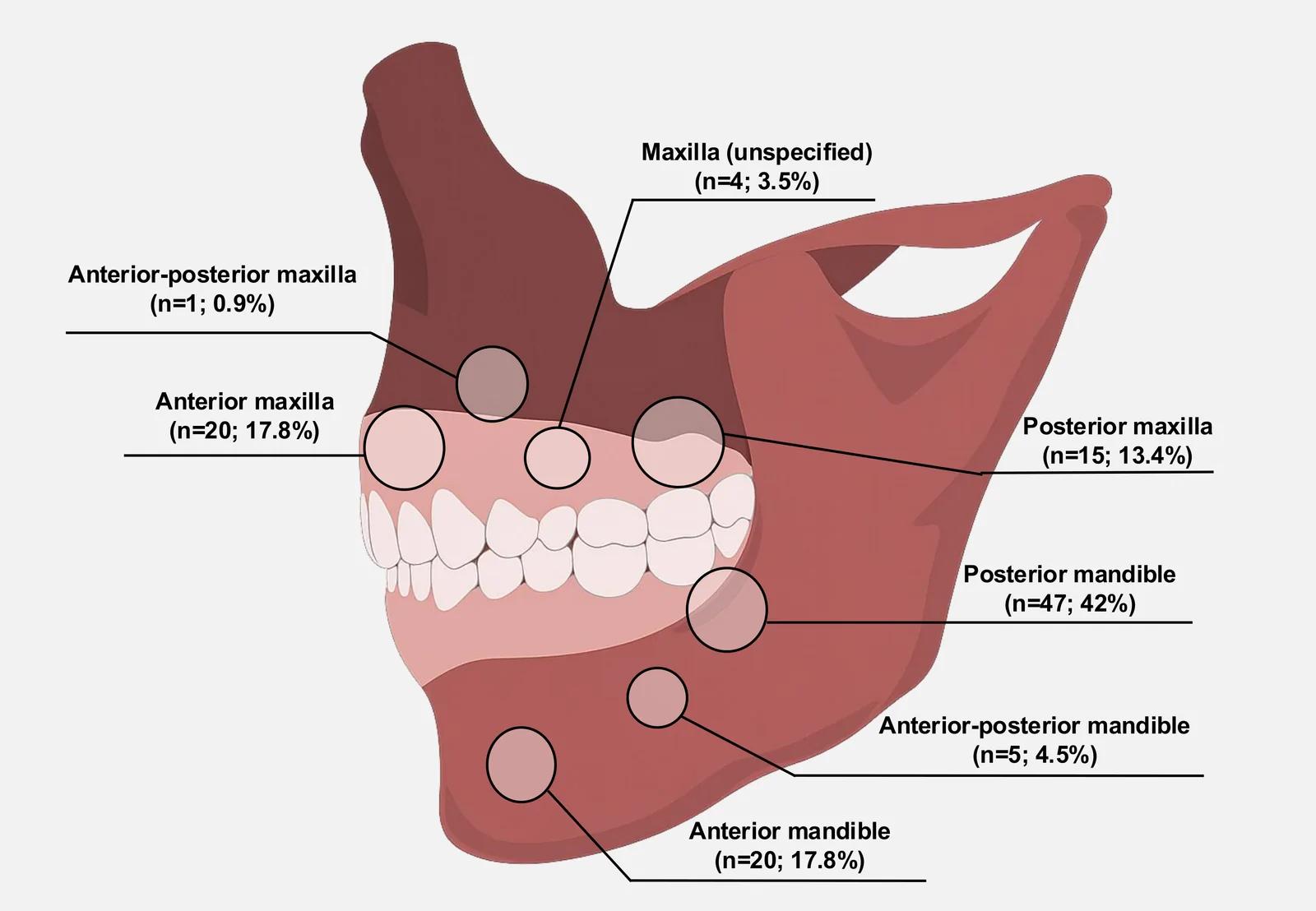
Fig. 2: Distribution of cemento-ossifying fibroma cases in the jaws.

**Figure 3:**
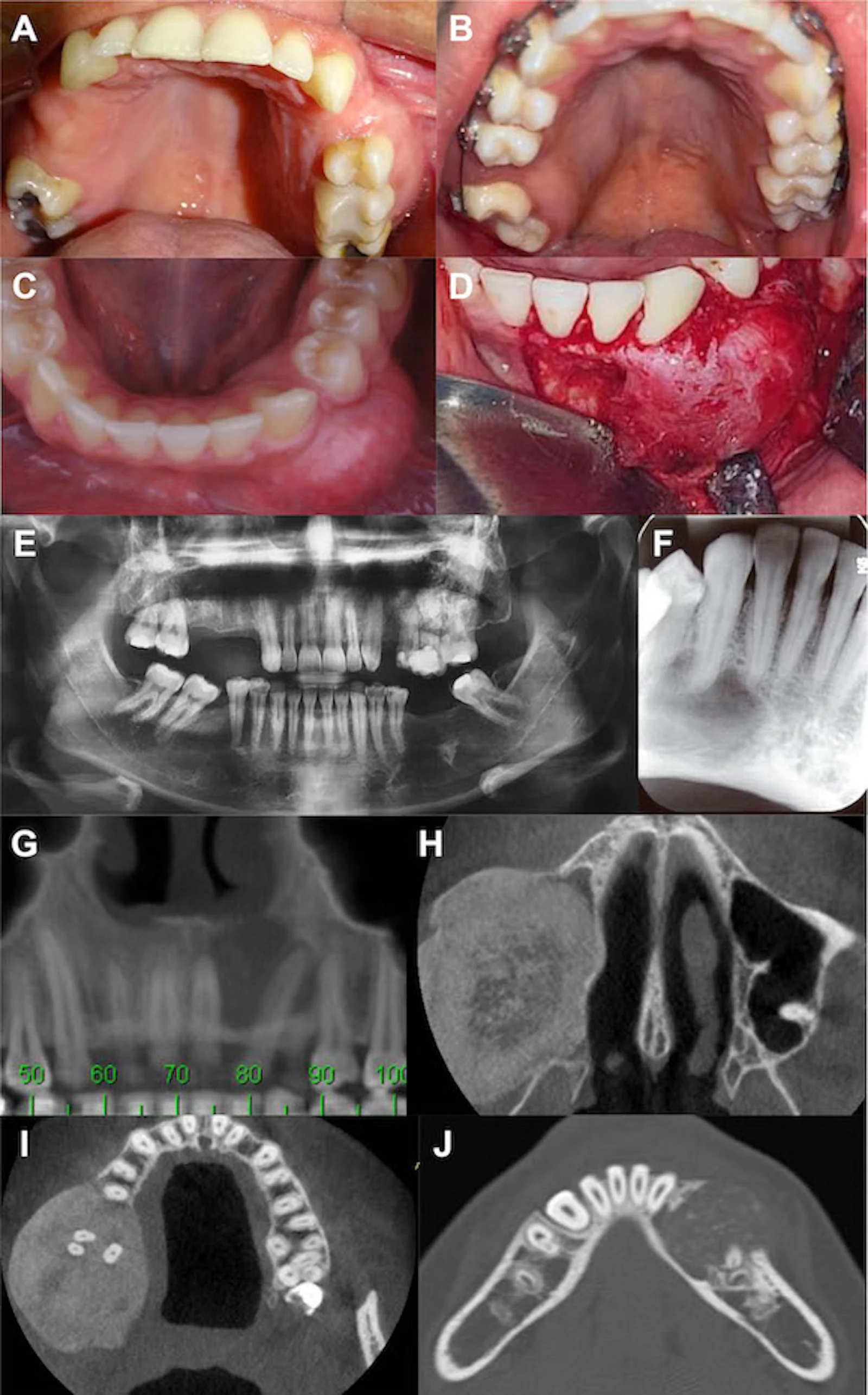
Fig. 3: Clinical and radiographic features of cemento-ossifying fibroma. (A) Intraoral view showing a swelling in the left posterior maxilla, normochromic, with a smooth surface and evident buccal and palatal expansion. (B) Maxillary case showing a swelling in the region of the right second and third molars. (C) Anterior mandibular lesion, normochromic, with an intact surface and noticeable buccal expansion and slight lingual expansion. (D) Intraoperative image revealing a well-circumscribed, encapsulated mass during surgical excision. (E) Panoramic radiograph of the lesion shown in (A), revealing a radiolucent area with multiple radiopaque foci, projected in the left posterior maxilla, close to the molar roots, and causing thinning of the alveolar cortex. (F) Periapical radiograph of a mandibular case depicting a well-circumscribed and partially corticated radiolucent lesion located apical to the roots of the right lateral incisor, canine, and first premolar. Note the loss of continuity of the lamina dura in the apical third of the canine. (G) Panoramic computed tomography (CT) view revealing a hypodense lesion in the left anterior maxilla with a sharply corticated border. The lesion causes divergence of the canine root. (H) Coronal CT scan showing a mixed-density lesion within the right maxillary sinus, causing expansion of the buccal cortex and slight expansion and thinning of the medial cortex toward the nasal cavity. (I) Axial CT scan demonstrating a mixed-density lesion located in the posterior region of the right maxilla, with noticeable expansion and thinning of both buccal and lingual cortices. (J) Axial CT scan showing an extensive and predominantly radiolucent mandibular lesion with thinning of the lingual cortex, as well as expansion and rupture of the buccal plate.

**Figure 4:**
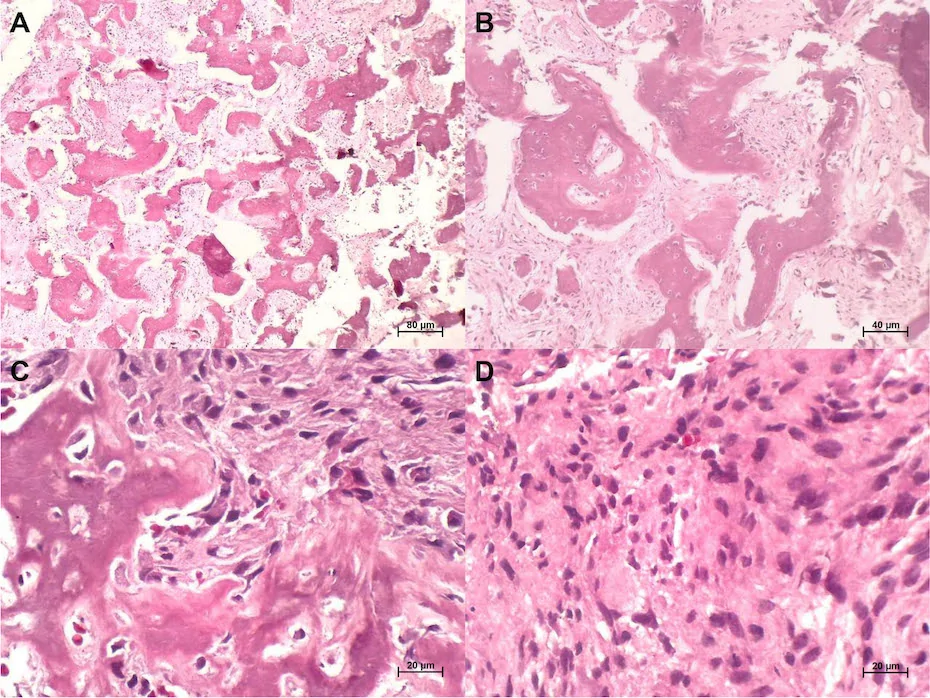
Fig. 4: Histopathological features of cemento-ossifying fibroma. (A) Low-power view showing a well-demarcated fibro-osseous tissue composed of a cellular fibrous stroma interspersed with numerous mineralized trabeculae. The mineralized component exhibits irregular woven bone and cementum-like deposits, with greater mineral density centrally. (B) Intermediate-power image demonstrating thick trabeculae of woven to lamellar bone, some of which show anastomosing and confluent patterns. Occasional osteoblastic rimming is evident, together with areas of osteoid deposition. (C-D) High-power views showing active stromal condensation around mineralized trabeculae. The stroma exhibits mild hypercellularity, but nuclei remain uniform without pleomorphism (hematoxylin and eosin staining: A, ×100 magnification; B, ×200 magnification; C-D, ×400 magnification).

## Data Availability

All research data supporting this publication are available within this manuscript and its supplemental material.
